# Cognitive capability and behavioral exposure in generative AI use: a dual-pathway model of perceived facilitation and integrity-related risk

**DOI:** 10.3389/fpsyg.2026.1876096

**Published:** 2026-07-09

**Authors:** Song Mu, Bo Wang, Juan Xiong, Yuqing Xie, Weijian Hu

**Affiliations:** 1 Guangzhou College of Technology and Business, Guangzhou, China; 2 Guangdong University of Finance & Economics, Guangzhou, China

**Keywords:** academic integrity, AI familiarity, generative artificial intelligence, measurement invariance, structural equation modelling

## Abstract

The rapid adoption of generative artificial intelligence (GenAI) has introduced new forms of cognitive support as well as potential behavioral risks in academic contexts. Rather than treating AI use as a purely homogeneous phenomenon, this study conceptualizes GenAI engagement through a psychological dual-pathway framework that explicitly separates capability-oriented AI familiarity from exposure-based AI use frequency. We examine how these distinct pathways map onto perceived cognitive facilitation, cognitive reliance, and integrity-related behavioral risk. Data were collected from two independent samples of university students (*N* = 407 and *N* = 228; total *N* = 635) and analyzed using structural equation modeling. Measurement invariance analyses supported configural, metric, and scalar invariance across the two samples, indicating that the latent constructs were interpreted in broadly comparable ways across cohorts. Results from the main sample indicated that AI familiarity was positively associated with perceived cognitive facilitation and negatively associated with integrity-related behavioral risk, whereas AI use frequency showed weaker and less consistent relationships with the outcome variables. However, these structural relationships were substantially weaker and largely non-significant in the replication sample, suggesting that the observed associations may be context-sensitive rather than uniformly stable across learning environments. The findings highlight an important distinction between cognitive capability and behavioral exposure in understanding students’ engagement with GenAI. Overall, the results suggest that students’ educational experiences with GenAI may depend less on simple usage frequency and more on their ability to critically understand, evaluate, and regulate AI-supported learning processes. These findings have implications for AI literacy development, academic integrity governance, and assessment design in higher education.

## 1 Introduction

The rapid development of generative artificial intelligence (GenAI) is reshaping higher education worldwide, creating new opportunities for learning support while simultaneously raising concerns regarding ethical use, transparency, academic integrity, and educational governance (UNESCO, 2023). Tools such as ChatGPT are increasingly used by university students to generate explanations, summarize academic materials, support writing, and provide immediate feedback during learning activities. As these systems become more accessible and embedded in everyday learning practices, they are beginning to influence not only how students study, but also broader discussions concerning independent thinking, responsible technology use, and the boundaries of acceptable AI-assisted academic behavior (Baidoo-Anu and Ansah, 2023; Kasneci et al., 2023). Consequently, understanding how students psychologically engage with GenAI has become an increasingly important issue in higher education research.

Recent educational and psychological research has shown growing interest in the role of GenAI in higher education, particularly in relation to learning engagement, cognitive support, assessment, and self-regulated learning processes (Lo et al., 2024; Zawacki-Richter et al., 2024). At the same time, concerns have emerged regarding excessive dependence on AI-generated content, reduced critical reflection, and inappropriate academic use of AI systems (Cotton et al., 2024; Kim et al., 2025). These debates suggest that GenAI should not be conceptualized simply as either a beneficial educational technology or a source of academic risk. Rather, its educational impact may depend on how students understand, evaluate, and regulate their engagement with AI-generated information. Accordingly, both supportive and risk-related dimensions of AI engagement require simultaneous consideration within a unified analytic framework.

This issue may be particularly important in digitally mediated and open learning environments, where students often rely more heavily on independent learning strategies and self-directed interaction with digital resources (Bozkurt et al., 2020). In such contexts, instructor supervision is typically less immediate, and students may need to make their own judgments regarding when AI assistance is appropriate, how generated information should be verified, and where the boundary lies between productive support and excessive dependence. Under these conditions, students’ understanding of AI tools may become as important as the tools themselves.

Existing studies have documented several potential educational benefits associated with GenAI use. AI-assisted systems may support idea generation, task organization, conceptual understanding, and learning efficiency (Dwivedi et al., 2023; Huang et al., 2024; Kasneci et al., 2023). Some students also report that AI tools help reduce academic pressure and facilitate learning-related self-management. However, other studies have raised concerns that extensive reliance on AI-generated content may weaken critical engagement, encourage passive learning habits, or increase opportunities for academically inappropriate behavior (Cotton et al., 2024; Kim et al., 2025). These mixed findings suggest that GenAI engagement involves both learning-supportive and risk-related dimensions.

Another issue concerns how student engagement with GenAI is conceptualized. Many existing studies operationalize AI engagement primarily through usage frequency. Although frequency captures behavioral exposure, it does not necessarily reflect whether students understand how to use AI critically, responsibly, or strategically. Two students may use GenAI equally often while differing substantially in their ability to evaluate AI-generated information, verify sources, or regulate dependence on generated outputs. As a result, frequency-based indicators may oversimplify the psychological processes underlying AI-supported learning behavior.

For this reason, perceived AI familiarity may represent a distinct and potentially more meaningful dimension of GenAI engagement. In the present study, AI familiarity refers to students’ perceived understanding of AI tools, including awareness of functions, practical experience, and confidence in navigating AI-supported learning situations. In broader educational technology research, familiarity and confidence with digital tools have been associated with lower adoption barriers and more active engagement in learning activities (Waluyo and Kusumastuti, 2024; Zhan and Yan, 2025). This perspective is also broadly consistent with emerging discussions of AI literacy, which emphasize critical evaluation, ethical awareness, and responsible integration of AI-generated information into learning processes rather than mere technological access (Wang et al., 2025; Xia et al., 2026). From this perspective, familiarity may function as a capability-oriented indicator associated with how students regulate and integrate AI support into learning activities.

At the same time, frequent use of AI systems should not automatically be interpreted as evidence of meaningful or reflective engagement. Students may use AI tools frequently for convenience, task completion, or efficiency without necessarily engaging critically with generated information. Recent discussions in self-regulated learning and educational technology research similarly suggest that educational outcomes may depend less on technological exposure itself and more on how learners evaluate, monitor, and strategically apply technological support during learning processes (Lo et al., 2024; Rizun et al., 2026). Distinguishing between AI familiarity and AI use frequency may therefore help clarify why previous studies have reported inconsistent findings regarding the educational effects of GenAI use.

The present study examines the relationships between two forms of GenAI engagement—AI familiarity and AI use frequency—and three outcome dimensions related to learning support and AI-related risk. AI familiarity is conceptualized as a capability-oriented indicator reflecting students’ perceived understanding of AI systems, whereas AI use frequency is conceptualized as an exposure-oriented indicator reflecting how often students engage with GenAI in learning-related contexts. The study further examines three outcome dimensions: perceived cognitive facilitation, cognitive reliance on AI, and integrity-related behavioral risk.

Rather than proposing a wholly new theory of GenAI engagement, the present study offers an integrative analytic perspective that brings together insights from AI literacy research, digital competence frameworks, educational technology adoption research, and self-regulated learning theory. The distinction between familiarity and frequency is theoretically meaningful because students may use AI frequently without necessarily understanding how to evaluate or regulate AI-generated content, whereas students with greater familiarity may engage with AI in more reflective and controlled ways.

Methodologically, the study adopts a two-sample strategy using independently collected datasets processed with identical data-screening procedures. Rather than treating replication as a simple confirmation exercise, the study distinguishes between measurement stability and structural consistency across samples. This distinction is particularly relevant in rapidly evolving GenAI contexts, where institutional guidance, assessment practices, disciplinary expectations, and student norms may differ across learning environments (Makel and Plucker, 2014). Accordingly, the study examines not only whether the proposed constructs can be measured consistently across cohorts, but also whether the structural relationships among these constructs remain stable across learning contexts.

## 2 Hypotheses

Recent studies suggest that GenAI tools are often perceived by students as useful for explanation seeking, task organization, writing support, and learning assistance (Dwivedi et al., 2023; Kasneci et al., 2023). However, the educational value of AI may depend not only on whether students use these systems, but also on whether they possess sufficient understanding to evaluate and regulate AI-assisted learning processes. Students who are more familiar with AI tools may be better able to formulate effective prompts, critically assess generated information, verify outputs, and integrate AI support into learning activities in a more reflective manner. Research on AI literacy and digital competence similarly emphasizes that meaningful educational use of AI requires more than technological access alone; it also involves critical awareness, evaluative ability, and responsible engagement with AI-generated content (Long and Magerko, 2020; Wang et al., 2025; Xia et al., 2026). Accordingly, students with greater perceived AI familiarity may be more likely to experience AI as cognitively supportive rather than merely convenient.

At the same time, greater familiarity with AI systems may also relate to lower levels of problematic AI-related behavior. Students who better understand the limitations, risks, and appropriate boundaries of AI-generated content may be less likely to rely uncritically on AI outputs or engage in academically inappropriate AI-assisted practices. Prior discussions of responsible AI use similarly suggest that critical understanding and ethical awareness may support self-regulation and reduce problematic forms of technology-assisted academic behavior (Cotton et al., 2024; Long and Magerko, 2020). Research on academic integrity in digital learning environments has likewise emphasized the importance of self-regulation, ethical judgment, and clearer understanding of acceptable technology use (Bin-Nashwan et al., 2023; Kangwa et al., 2025). Consequently, students with higher perceived AI familiarity may be better positioned to maintain reflective control over AI-assisted learning processes and avoid problematic academic behaviors.

However, the relationship between familiarity and AI-related risk may not be entirely straightforward. Students who understand AI systems may still rely heavily on them under conditions such as time pressure, academic workload, or ambiguous assessment expectations. Accordingly, these relationships are examined empirically rather than assumed to be uniformly strong across contexts.

In contrast, AI use frequency primarily reflects behavioral exposure rather than reflective understanding. Frequent AI use does not necessarily imply critical engagement, strategic regulation, or responsible application. Students may use AI systems frequently for convenience or efficiency while differing substantially in how they evaluate AI-generated information and regulate dependence on generated responses. Recent research in educational technology and self-regulated learning similarly suggests that educational outcomes depend less on usage intensity alone than on learners’ ability to evaluate and strategically apply technological support during learning activities (Lo et al., 2024; Rizun et al., 2026; Zhan and Yan, 2025). Accordingly, AI use frequency is expected to show weaker and less consistent associations with learning-supportive and risk-related outcomes than AI familiarity. Rather than assuming that more frequent AI use necessarily leads to positive or negative educational consequences, the present study conceptualizes frequency primarily as an indicator of behavioral exposure to AI-supported learning environments.

Beyond direct individual-level pathways, the relationships between GenAI engagement and learning outcomes may also vary across educational contexts. Institutional guidance, assessment practices, disciplinary expectations, and localized norms regarding AI use may shape how students integrate AI into learning activities (Zawacki-Richter et al., 2024). Accordingly, even if the measurement structure remains stable across cohorts, the structural relationships among the variables may vary across learning environments.

Based on these considerations, the study proposes the following hypothesized framework:

*H1*: AI familiarity will be positively associated with perceived cognitive facilitation.

*H2a*: AI familiarity will be negatively associated with cognitive reliance on AI.

*H2b*: AI familiarity will be negatively associated with integrity-related behavioral risk.

*H3*: The study explores whether the structural relationships among GenAI engagement and the outcome variables remain stable across the two samples, even if the measurement structure demonstrates acceptable cross-sample equivalence.

## 3 Methods

### 3.1 Research design

This study employed a cross-sectional survey design to examine how different forms of GenAI engagement relate to perceived learning-supportive and risk-related outcomes among university students. Consistent with the theoretical framework proposed in the Introduction, the study distinguished between two forms of GenAI engagement: AI familiarity and AI use frequency. AI familiarity was conceptualized as a capability-oriented indicator reflecting students’ perceived understanding of AI tools and confidence in navigating AI-supported learning situations, whereas AI use frequency was conceptualized as an exposure-oriented indicator reflecting how often students used GenAI in learning-related contexts.

Three outcome dimensions were modeled as latent variables: perceived cognitive facilitation, cognitive reliance on AI, and integrity-related behavioral risk. Perceived cognitive facilitation reflected students’ perceptions that AI supports understanding, learning efficiency, academic development, and learning-related self-management. Cognitive reliance on AI captured concerns regarding excessive dependence on AI-generated outputs and reduced independent thinking. Integrity-related behavioral risk referred to self-reported tendencies toward problematic academic AI use, including weak attribution practices and passive AI-assisted learning behaviors.

**Figure 1:**
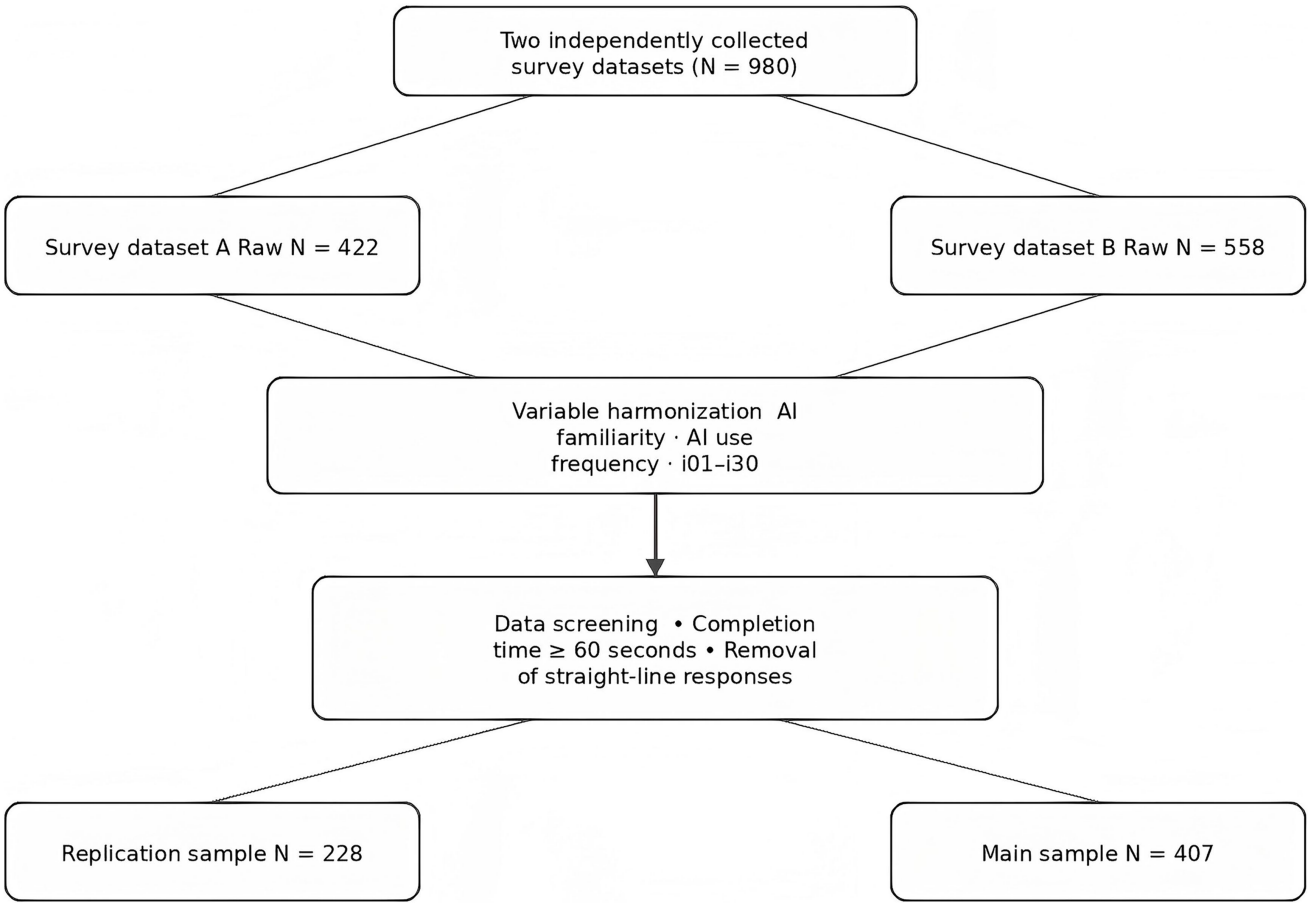
Study design and analytic workflow. The figure summarizes the overall workflow of the study, including dataset collection, variable harmonization, data screening, and the final analytic samples used for SEM analyses.

To strengthen the robustness of the findings, the study adopted a two-sample analytic strategy using two independently collected datasets. Rather than randomly splitting a single dataset, the two datasets were processed using identical data-screening procedures and analyzed separately. This approach allowed the study to distinguish between measurement stability and structural consistency across samples, which is particularly relevant in rapidly evolving GenAI contexts where institutional guidance, assessment expectations, and student learning practices may differ across environments (Makel and Plucker, 2014). Accordingly, the two-sample strategy was intended not only to evaluate the reproducibility of the measurement structure, but also to examine whether the proposed structural relationships remained stable across different student contexts. The overall study workflow is presented in Figure 1.

### 3.2 Participants and data screening

Participants were undergraduate students recruited through course-based distribution, institutional communication channels, and online learning platforms. Participation was voluntary, and respondents were informed that the survey was conducted for academic research purposes only. No personally identifiable information was collected, and all analyses were conducted using anonymized aggregate data.

The original datasets contained 980 responses (Dataset 1: *n* = 558; Dataset 2: *n* = 422). Only variables shared across both datasets were retained for analysis, including AI familiarity, AI use frequency, completion time, and 30 Likert-type items measuring the three latent outcome dimensions. To improve data quality, two screening procedures were applied consistently across both datasets. First, responses completed in less than 60 s were removed because they were considered unlikely to reflect meaningful engagement with the questionnaire. Second, straight-line responses showing identical answers across all items were excluded. After screening, 407 valid responses were retained in the main sample and 228 in the replication sample, resulting in a total analytic sample of 635 participants. Demographic characteristics of the two samples are summarized in Table 1. Both samples were composed primarily of students from business- and economics-related disciplines, and female participants represented the majority in both datasets.

**Table 1:** Demographic characteristics of the analytic sample.

Sample	Gender distribution	Major group distribution
Main (*N* = 407)	Male 32.7%; Female 67.3%	Business/Econ 72.3%; Languages 15.6%; Engineering 4.2%; Other 7.9%
Validation (*N* = 228)	Male 21.5%; Female 78.5%	Business/Econ 86.4%; Languages 8.3%; Design/Art 2.6%; Other 2.7%

Gender categories are retained as anonymized survey categories. The sample was concentrated primarily in business-related undergraduate contexts.

### 3.3 Measures

All substantive items used five-point Likert-type response scales ranging from 1 (strongly disagree) to 5 (strongly agree).

### 3.4 AI familiarity

AI familiarity was assessed using a self-reported item reflecting students’ perceived understanding of AI tools, including awareness of functions, practical experience, and confidence in using AI in learning-related situations. Consistent with the conceptual framework of the study, AI familiarity was treated as a capability-oriented indicator rather than a direct measure of objective AI literacy or technical competence. The construct was intended to capture students’ subjective sense of cognitive readiness for engaging with AI-supported learning rather than formal technological expertise. Following methodological recommendations for broad, evaluative cognitive dispositions, a well-anchored single-item design is psychometrically efficient and minimizes respondent fatigue without sacrificing predictive validity (Wanous et al., 1997).

### 3.5 AI use frequency

AI use frequency measured how often students reported using GenAI tools in learning-related contexts and was treated as an indicator of behavioral exposure to AI-supported learning activities. Because behavioral exposure represents an objective, unidimensional temporal count rather than a complex latent psychological construct, a single-item frequency metric serves as an empirically justified and reliable standard in educational technology research (Hair et al., 2021; Kline, 2023). In the present study, use frequency was conceptualized primarily as an exposure-based behavioral measure rather than as an indicator of reflective or strategic AI engagement.

### 3.6 Perceived cognitive facilitation

Perceived cognitive facilitation was measured using items i01–i16. These items capture multiple but closely related aspects of AI-supported learning, including support for conceptual understanding, knowledge expansion, learning efficiency, academic development, and learning-related self-management.

The decision to model these items as a single latent construct was based on both conceptual and empirical considerations. Conceptually, students’ interactions with GenAI often combine explanation seeking, planning support, and self-regulated learning processes within the same learning activity. For example, students using AI to clarify academic content may simultaneously receive support related to organizing tasks or managing learning strategies. Accordingly, these experiences were treated as components of a broader facilitative learning perception rather than as entirely separate dimensions. Empirically, the items demonstrated strong internal consistency and convergent validity, supporting a parsimonious latent representation. This broader facilitation construct was therefore intended to reflect students’ integrated perception of AI-supported learning experiences rather than narrowly isolated learning functions.

### 3.7 Cognitive reliance on AI

Cognitive reliance on AI was measured using items i17–i22 and reflected concerns regarding excessive dependence on AI-generated outputs, reduced independent thinking, and uncritical acceptance of AI-generated information.

### 3.8 Integrity-related behavioral risk

Integrity-related behavioral risk was measured using items i23–i28 and i30. These items captured self-reported tendencies toward problematic academic AI use, including weak attribution practices, passive AI-assisted learning, and potentially inappropriate dependence on generated content. Item i29 was excluded because it reflected a normative evaluative statement rather than a behavioral tendency.

**Figure 2:**
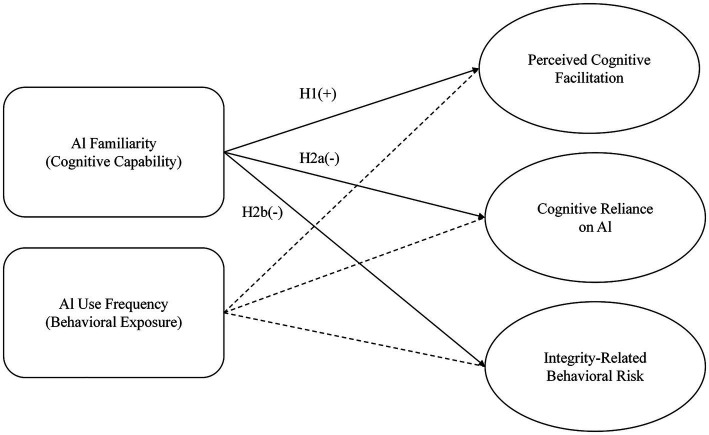
Conceptual model and hypothesized relationships. The conceptual model illustrates the hypothesized relationships among AI familiarity, AI use frequency, perceived cognitive facilitation, cognitive reliance on AI, and integrity-related behavioral risk.

The conceptual SEM framework and hypothesized relationships are illustrated in Figure 2.

### 3.9 Data analysis

The analyses were conducted in R using the lavaan package (Rosseel, 2012). Structural equation modeling (SEM) was selected because it allows latent constructs and measurement error to be modeled simultaneously while examining relationships among multiple observed and latent variables (Kline, 2023).

Model estimation employed robust maximum likelihood estimation (MLR) to reduce sensitivity to non-normality. Missing data were handled using Full Information Maximum Likelihood (FIML) procedures (Enders, 2022). Latent variables were standardized using the *std.lv* = *TRUE* specification.

First, harmonized variables shared across the two datasets were extracted and screened using identical quality-control procedures. Second, reliability and convergent validity were evaluated using Cronbach’s alpha, composite reliability (CR), and average variance extracted (AVE). Third, SEM models were estimated separately for the main and replication samples to evaluate the structural relationships proposed in H1, H2a, and H2b. Fourth, measurement invariance testing was conducted across samples using configural, metric, and scalar invariance models to evaluate whether the latent constructs operated similarly across cohorts. Establishing measurement invariance was necessary before interpreting potential differences in structural relationships across the two samples. Fifth, the potential risk of common method bias was evaluated using complementary exploratory and confirmatory procedures. Harman’s single-factor tests were executed independently for the primary and replication cohorts to evaluate variance accumulation, which was further cross-validated through a competitive single-factor confirmatory factor analysis (CFA) estimated in the main sample. Finally, robustness checks were conducted using gender-controlled SEMs and composite-score regression models (Hair et al., 2021).

Model fit was evaluated using multiple indices, including the Comparative Fit Index (CFI), Tucker–Lewis Index (TLI), Root Mean Square Error of Approximation (RMSEA), Standardized Root Mean Square Residual (SRMR), and chi-square statistics (χ^2^). Following commonly used recommendations, changes of |ΔCFI| < 0.010 and |ΔRMSEA| < 0.015 were interpreted as evidence supporting measurement invariance across samples (Chen, 2007; Cheung and Rensvold, 2002).

Because the proposed model integrated conceptually broad educational and psychological dimensions, model evaluation emphasized both statistical fit and theoretical interpretability. Accordingly, emphasis was placed not only on incremental fit indices but also on conceptual coherence, residual-based fit indices, and cross-sample interpretability.

### 3.10 Common method bias assessment

Because all substantive variables were collected using self-reported survey responses, common method bias was assessed using two complementary procedures. First, Harman’s single-factor test was conducted to examine whether a single unrotated factor accounted for the majority of covariance among the substantive items. Second, a single-factor CFA model, in which all substantive items were constrained to load onto one common latent factor, was estimated and compared with the proposed three-factor measurement structure. These analyses were conducted using the substantive items retained in the final measurement model. Using both exploratory and confirmatory procedures provided a more rigorous evaluation of whether the observed associations were likely to reflect substantive relationships rather than shared method variance alone.

## 4 Results

### 4.1 Reliability and convergent validity

The reliability and convergent validity of the three-factor measurement framework were first evaluated in the main sample. As shown in Table 2, all latent constructs demonstrated satisfactory psychometric properties.

**Table 2:** Reliability and convergent validity of the three-factor model (main sample).

**Construct**	**Items**	**Cronbach’s *α***	**CR**	**AVE**
Perceived cognitive facilitation	i01–i16	0.954	0.956	0.581
Cognitive reliance on AI	i17–i22	0.927	0.929	0.688
Integrity-related behavioral risk	i23–i28, i30	0.940	0.939	0.690

CR = composite reliability; AVE = average variance extracted.

Cronbach’s alpha values ranged from 0.927 to 0.954, indicating high internal consistency. Composite reliability (CR) values ranged from 0.929 to 0.956, exceeding the commonly recommended threshold of 0.70, while average variance extracted (AVE) values ranged from 0.581 to 0.690, supporting acceptable convergent validity (Hair et al., 2021). Overall, these findings suggest that the measurement structure showed adequate reliability and construct coherence for subsequent SEM analyses.

### 4.2 Structural model results in the main sample

**Figure 3:**
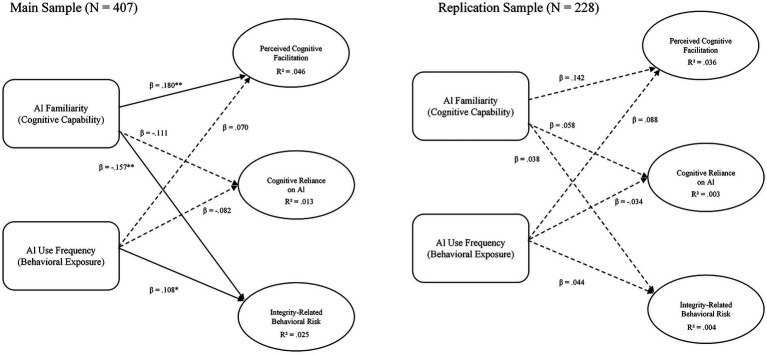
Final SEM results across the main and replication samples. Values represent standardized structural coefficients (*β*). Solid lines indicate significant paths (*p < 0.05, **p < 0.01); dashed lines represent non-significant relations.

The SEM results for the main sample are presented in Table 3 and Figure 3. Consistent with Hypothesis 1, AI familiarity was positively associated with perceived cognitive facilitation (*β* = 0.180, *p* = 0.001). Students who reported greater familiarity with GenAI tools were more likely to perceive AI as helpful for understanding content, organizing learning tasks, and supporting academic development. This finding is broadly consistent with the capability-oriented interpretation of AI familiarity proposed in the theoretical framework.

**Table 3:** Structural paths in the main sample.

Outcome	Predictor	Standardized β	*p*
Perceived cognitive facilitation	AI use frequency	0.070	0.190
Perceived cognitive facilitation	AI familiarity	0.180	0.001
Cognitive reliance on AI	AI use frequency	0.082	0.140
Cognitive reliance on AI	AI familiarity	−0.111	0.066
Integrity-related behavioral risk	AI use frequency	0.108	0.049
Integrity-related behavioral risk	AI familiarity	−0.157	0.008

With respect to Hypothesis 2b, AI familiarity also showed a negative association with integrity-related behavioral risk (*β* = −0.157, *p* = 0.008). The association between AI familiarity and cognitive reliance on AI (Hypothesis 2a) was also negative, although the path did not reach conventional statistical significance (*β* = −0.111, *p* = 0.066). These findings suggest that students who perceived themselves as more familiar with AI tools tended to report fewer problematic AI-related academic tendencies, although familiarity alone may not fully reduce perceptions of cognitive dependence. Accordingly, the findings provide only partial support for the proposed relationship between AI familiarity and risk-related outcomes.

In contrast, AI use frequency showed weaker and less consistent relationships with the three outcomes. The paths from use frequency to perceived cognitive facilitation (*β* = 0.070, *p* = 0.190) and cognitive reliance on AI (*β* = 0.082, *p* = 0.140) were non-significant. The path from use frequency to integrity-related behavioral risk was positive but small in magnitude (*β* = 0.108, *p* = 0.049). Taken together, these findings generally supported the conceptual distinction between capability-oriented familiarity and simple behavioral exposure.

### 4.3 Model fit and replication results

Model fit statistics for both datasets are shown in Table 4. Across the two samples, the SEM models demonstrated comparable fit patterns. RMSEA and SRMR values were within commonly accepted ranges, whereas CFI and TLI values remained below the conventional 0.90 criterion. Following recent SEM recommendations emphasizing the joint interpretation of multiple indices rather than strict cutoff reliance, the overall fit was interpreted as marginal-to-acceptable rather than poor (Kline, 2023).

**Table 4:** Model fit indices and explained variance.

Sample	CFI	TLI	RMSEA	SRMR	χ^2^	df	Facilitation *R*^2^	Reliance *R*^2^	Risk *R*^2^
Main sample	0.851	0.838	0.077	0.055	1444.507	426	0.046	0.013	0.025
Replication sample	0.849	0.836	0.078	0.053	1012.473	426	0.036	0.003	0.004

The relatively modest CFI and TLI values may partly reflect the broad facilitation construct and the relatively large number of observed indicators included in the model. Because the facilitation construct integrated several conceptually related aspects of AI-supported learning, the model prioritized conceptual coherence over post-hoc statistical simplification.

The explained variance values were relatively small. In the main sample, AI familiarity and AI use frequency explained 4.6% of the variance in perceived cognitive facilitation, 1.3% of the variance in cognitive reliance on AI, and 2.5% of the variance in integrity-related behavioral risk. These findings indicate that the observed relationships should be interpreted as modest associations rather than strong explanatory effects. This pattern also suggests that broader contextual and instructional factors likely contribute substantially to students’ AI-related learning experiences and behaviors.

### 4.4 Replication sample results

The structural relationships observed in the main sample were not fully reproduced in the replication sample (Table 5). Although the direction of the association between AI familiarity and perceived cognitive facilitation remained positive, the effect size was smaller and did not reach statistical significance (*β* = 0.142, *p* = 0.087). Similarly, the remaining structural paths were weak and non-significant.

**Table 5:** Structural paths in the replication sample.

Outcome	Predictor	Standardized β	*p*
Perceived cognitive facilitation	AI use frequency	0.088	0.244
Perceived cognitive facilitation	AI familiarity	0.142	0.087
Cognitive reliance on AI	AI use frequency	−0.034	0.637
Cognitive reliance on AI	AI familiarity	0.058	0.458
Integrity-related behavioral risk	AI use frequency	0.044	0.522
Integrity-related behavioral risk	AI familiarity	0.038	0.633

Importantly, these weaker replication results should not necessarily be interpreted as evidence of model failure. Rather, the findings suggest that the relationships between GenAI engagement and learning-related outcomes may be context-sensitive and influenced by variations in instructional practices, assessment expectations, disciplinary cultures, or institutional guidance regarding AI use. This interpretation is consistent with the study’s theoretical distinction between measurement stability and structural consistency across cohorts.

More specifically, the replication findings provide partial support for Hypothesis 3, indicating that although the latent constructs were measured similarly across the two samples, the structural relationships among these constructs were less stable across contexts. The findings therefore suggest that the relationships between AI familiarity and learning-related outcomes may vary across educational contexts.

### 4.5 Measurement invariance across samples

Measurement invariance testing was conducted to evaluate whether the three-factor measurement structure operated similarly across the two samples. The results are presented in Table 6. The configural model established a common factor structure across groups. Metric invariance constrained factor loadings to equality, and scalar invariance additionally constrained item intercepts.

**Table 6:** Measurement invariance across samples.

Model	χ^2^	df	CFI	TLI	RMSEA	SRMR	ΔCFI	ΔRMSEA
Configural	2275.656	748	0.850	0.837	0.080	0.057	—	—
Metric	2328.581	774	0.848	0.840	0.080	0.060	−0.003	−0.001
Scalar	2424.193	800	0.841	0.838	0.080	0.062	−0.007	<0.001

Changes in CFI and RMSEA remained within recommended thresholds across all comparisons. From configural to metric invariance, ΔCFI = −0.003 and ΔRMSEA = −0.001. From metric to scalar invariance, ΔCFI = −0.007 and ΔRMSEA < 0.001, both within the commonly recommended cutoff criteria proposed by Chen (2007). These findings support acceptable cross-sample measurement equivalence. Accordingly, the latent constructs appeared to retain comparable psychometric meaning across the two cohorts despite the weaker replication of structural relationships.

Importantly, measurement equivalence should not be interpreted as evidence that the structural pathways themselves were invariant across contexts. Rather, the invariance results indicate that students in the two samples interpreted the measurement constructs in broadly similar ways, while the relationships among these constructs may still vary depending on contextual learning conditions. This distinction between measurement stability and structural variability represents one of the central analytic contributions of the present study.

### 4.6 Common method bias and robustness checks

Because all primary variables were collected from the same respondents within a single time frame, the potential risk of common method variance was evaluated using both exploratory and confirmatory approaches. Harman’s single-factor tests indicated that the largest unrotated factor accounted for 38.88% of the total variance in the main sample and 35.22% in the replication sample. Both values were below the commonly referenced 40% threshold, suggesting that common method variance was unlikely to represent a dominant source of covariance.

To further evaluate this issue, a single-factor CFA model was estimated in the main sample. The single-factor model demonstrated poor fit, χ^2^(377) = 5772.54, CFI = 0.430, TLI = 0.386, RMSEA = 0.150, and SRMR = 0.248. By comparison, the proposed three-factor measurement model demonstrated substantially better fit, χ^2^(374) = 1747.21, CFI = 0.855, TLI = 0.842, RMSEA = 0.076, and SRMR = 0.054. This substantial improvement in fit supports the interpretation that the observed covariance structure was unlikely to be explained by a single common latent factor alone.

Additional SEM models including gender as a control variable produced substantively similar findings. In the main sample, AI familiarity remained positively associated with perceived cognitive facilitation (*β* = 0.176, *p* = 0.002) and negatively associated with integrity-related behavioral risk (*β* = −0.153, *p* = 0.010). Gender itself exhibited no statistically significant relationships with any of the latent outcomes.

Furthermore, alternative composite-score regression analyses yielded highly similar patterns. In the main sample, AI familiarity remained positively associated with perceived cognitive facilitation (*β* = 0.174, *p* = 0.001) and negatively associated with integrity-related behavioral risk (*β* = −0.153, *p* = 0.004), whereas the replication sample again demonstrated weaker structural relationships overall. Taken together, these robustness checks suggest that the primary findings were not driven by gender composition or by the specific latent-variable estimation procedures used in the SEM analyses.

## 5 Discussion

The present study examined the relationships between two forms of GenAI engagement—capability-oriented AI familiarity and exposure-oriented AI use frequency—and three learning-supportive and risk-related outcomes among university students. Overall, the findings suggest that students’ perceived understanding of AI tools may be more relevant than simple usage frequency in explaining how students interpret and regulate AI-supported learning experiences. At the same time, the relatively modest effect sizes and weaker replication results indicate that these relationships should be interpreted cautiously and understood as context-sensitive rather than universally stable.

The clearest finding was the positive association between AI familiarity and perceived cognitive facilitation in the main sample. Students who reported greater familiarity with GenAI were more likely to perceive AI as helpful for understanding content, organizing learning tasks, improving learning efficiency, and supporting academic development. This finding is consistent with prior research suggesting that the educational value of AI depends not only on technological access, but also on users’ ability to engage with AI critically and purposefully (Kasneci et al., 2023; Xiao et al., 2024). It also aligns with research on AI literacy and digital competence emphasizing evaluative awareness, reflective judgment, and strategic use of AI-supported learning tools (Long and Magerko, 2020; Wang et al., 2025). From an educational psychology perspective, AI familiarity may therefore reflect a form of cognitive readiness that supports more reflective engagement with AI-assisted learning.

The results also showed that AI familiarity was negatively associated with integrity-related behavioral risk in the main sample. Students who perceived themselves as more familiar with AI tools reported fewer tendencies toward problematic AI-assisted academic behavior, including passive AI-supported learning and weak attribution practices. This finding is consistent with research suggesting that responsible AI use involves not only technological access, but also ethical awareness, critical evaluation, and self-regulation (Cotton et al., 2024; Long and Magerko, 2020). However, the relationship between AI familiarity and cognitive reliance on AI was weaker and did not reach conventional significance levels. This suggests that familiarity alone may not fully protect against all forms of AI-related dependence. Students may understand the limitations of AI systems while still relying heavily on them under conditions such as time pressure, academic workload, or unclear assessment expectations.

In contrast, AI use frequency demonstrated weak and inconsistent relationships across the outcome variables. Frequency was not significantly associated with perceived cognitive facilitation or cognitive reliance in the main sample, and its association with integrity-related behavioral risk was small in magnitude. Similar patterns emerged in the replication sample, where all frequency-related paths were non-significant. These findings suggest that frequent AI use alone should not be interpreted as evidence of meaningful, reflective, or educationally beneficial engagement with GenAI. This interpretation is consistent with recent research indicating that educational outcomes depend less on usage intensity itself and more on the quality of learner engagement, strategic regulation, and critical evaluation during technology-supported learning processes (Jayasinghe et al., 2026; Lo et al., 2024; Rizun et al., 2026).

An important contribution of the study concerns the distinction between measurement stability and structural consistency across samples. Measurement invariance analyses demonstrated acceptable configural, metric, and scalar invariance, indicating that the latent constructs retained broadly comparable psychometric meanings across the two cohorts. However, the structural paths observed in the main sample became substantially weaker and largely non-significant in the replication sample. These findings suggest that measurement stability does not necessarily imply structural stability across educational contexts. Rather, the relationships between GenAI engagement and educational outcomes may be influenced by contextual factors such as assessment practices, instructor guidance, disciplinary expectations, and institutional norms regarding AI use (Zawacki-Richter et al., 2024).

The broad facilitation construct used in the study also merits discussion. The construct integrated multiple aspects of perceived AI-supported learning, including conceptual understanding, planning support, learning efficiency, and self-management. This approach reflects the integrated nature of students’ AI-supported learning experiences, where explanation, planning, and self-regulation often occur simultaneously. Although this conceptual breadth may have contributed to the relatively modest incremental fit indices, retaining the broader construct helped preserve theoretical coherence across samples.

From a practical perspective, the findings suggest that educational responses to GenAI should move beyond simple access and usage metrics and place greater emphasis on students’ capability for reflective and responsible AI engagement. Students may benefit from guidance on evaluating AI-generated outputs, verifying information accuracy, understanding appropriate use boundaries, and reflecting critically on the role of AI in learning processes. This may be particularly important in digitally mediated learning environments, where students often rely more heavily on self-regulated learning strategies and independent interaction with digital tools (Baidoo-Anu and Ansah, 2023; Bozkurt et al., 2020). Overall, the findings suggest that the educational implications of GenAI may depend less on technological exposure itself than on the psychological and contextual processes shaping how students engage with AI-supported learning.

## 6 Practical implications

Several practical implications emerge from the present findings.

First, the results suggest that higher education institutions should place greater emphasis on students’ understanding of AI tools rather than focusing primarily on usage frequency. Across analyses, AI familiarity showed more consistent associations with positive learning-related perceptions and lower integrity-related behavioral risk than simple AI use frequency. This finding suggests that the educational value of GenAI may depend less on technological exposure alone and more on students’ ability to critically evaluate, regulate, and appropriately integrate AI into learning processes. Accordingly, universities may benefit from prioritizing capability-oriented AI literacy development, including critical judgment, ethical awareness, and reflective use of AI-supported learning tools (Long and Magerko, 2020; Xiao et al., 2024).

Second, the findings highlight the importance of combining capability development with institutional guidance. As GenAI becomes increasingly integrated into higher education, students may encounter uncertainty regarding appropriate AI-assisted learning practices, disclosure expectations, and acceptable use boundaries. Because the observed relationships varied across samples, institutional responses to GenAI should also consider disciplinary context, assessment practices, and local learning environments rather than relying solely on generalized policies. Previous research similarly suggests that effective AI integration requires not only technological access, but also governance structures that support ethical and reflective learning practices (Cotton et al., 2024; Kasneci et al., 2023).

Third, the study has implications for course and assessment design. Traditional assessment formats that rely heavily on take-home written work may become increasingly vulnerable to uncritical AI-assisted completion. Instructors may therefore need to incorporate assessment approaches that emphasize reasoning processes, oral explanation, source evaluation, reflective justification, and transparent acknowledgment of AI-assisted work. Such approaches may encourage more active engagement with learning materials rather than passive dependence on generated outputs.

Finally, the findings may be particularly relevant for digitally mediated and open learning environments, where students often rely more heavily on self-regulated learning strategies and independent interaction with digital technologies (Bozkurt et al., 2020). Under these conditions, the educational impact of GenAI is likely to depend not only on technological availability but also on students’ capacity for reflective and responsible self-regulation. AI literacy training, reflective-use activities, and explicit discussion of AI limitations may therefore become increasingly important components of higher education.

Overall, the findings suggest that educational responses to GenAI should move beyond simple exposure-based perspectives of AI use and place greater emphasis on students’ critical, reflective, and responsible engagement with AI-supported learning.

## 7 Limitations and future directions

Several limitations should be considered when interpreting the findings.

First, the study relied on cross-sectional self-reported survey data, which limits causal inference and prevents the observed relationships from being interpreted as definitive directional effects. Although Harman’s single-factor tests and the single-factor CFA comparison suggested that common method variance was unlikely to fully account for the findings, common method bias cannot be completely ruled out. Future research should therefore employ longitudinal, experimental, or mixed-method designs to better examine how students’ AI familiarity and AI-related learning behaviors develop over time.

Second, AI familiarity was measured using a single self-report item and should therefore be interpreted as a proxy for perceived familiarity rather than a comprehensive measure of AI literacy or objective AI competence. Future studies should incorporate multidimensional and psychometrically validated AI literacy measures capturing areas such as prompt formulation, critical evaluation, and ethical awareness (Lintner, 2024; Liu et al., 2025).

Third, the explanatory power of the structural model was relatively modest. AI familiarity and AI use frequency explained only a limited proportion of variance in the outcome variables, suggesting that students’ AI-related behaviors are likely shaped by broader instructional, motivational, and institutional influences. Future studies should therefore incorporate additional contextual variables, including assessment design, institutional AI-use norms, learning motivation, and prior digital learning experience.

Fourth, although RMSEA and SRMR values were acceptable, the CFI and TLI values remained below conventional 0.90 thresholds. The broad facilitation construct may have contributed to lower incremental fit indices because it intentionally integrated multiple related aspects of AI-supported learning into a single latent variable. Nevertheless, the primary structural patterns remained broadly comparable across robustness checks, supporting cautious interpretation of the core findings.

Finally, the sample was concentrated primarily in business-related undergraduate contexts and was female-dominated within a single national setting. Accordingly, the findings should not be generalized uncritically to other disciplinary, institutional, or international contexts. Future research should utilize more diverse and cross-cultural samples to further evaluate the generalizability of the proposed framework.

## 8 Conclusion

This study examined both learning-supportive and risk-related aspects of GenAI engagement in higher education by distinguishing between capability-oriented AI familiarity and exposure-oriented AI use frequency within a unified analytical framework. The findings suggest that AI familiarity may be more informative than simple usage frequency in understanding students’ perceptions of AI-supported learning and integrity-related behavioral tendencies.

In the main sample, students with greater perceived familiarity reported stronger perceptions of cognitive facilitation and lower integrity-related behavioral risk, whereas AI use frequency demonstrated comparatively weak and inconsistent relationships with the outcome variables. These findings suggest that students’ engagement with GenAI in learning contexts may depend less on technological exposure alone and more on their ability to critically evaluate, regulate, and integrate AI-supported learning into academic processes.

At the same time, the weaker replication results indicate that these relationships may vary across educational contexts. Although the measurement structure remained broadly stable across samples, the structural pathways were less consistent, suggesting that institutional environments, assessment practices, and localized patterns of AI integration may shape how students engage with GenAI.

Overall, the findings highlight the importance of moving beyond exposure-based perspectives of AI use in higher education. As GenAI becomes increasingly integrated into university learning environments, educational institutions may need to place greater emphasis not only on access to AI technologies, but also on students’ critical, reflective, and responsible engagement with AI-supported learning.

## Data Availability

The datasets presented in this study can be found in online repositories. The names of the repository/repositories and accession number(s) can be found in the article/Supplementary material.
